# A Feature-Trajectory-Smoothed High-Speed Model for Video Anomaly Detection

**DOI:** 10.3390/s23031612

**Published:** 2023-02-02

**Authors:** Li Sun, Zhiguo Wang, Yujin Zhang, Guijin Wang

**Affiliations:** 1 Department of Electronic Engineering, Tsinghua University, Beijing 100084, China; 2 Shanghai AI Laboratory, Shanghai 200232, China

**Keywords:** anomaly detection, generation error, feature trajectory smoothness, surveillance video

## Abstract

High-speed detection of abnormal frames in surveillance videos is essential for security. This paper proposes a new video anomaly–detection model, namely, feature trajectory–smoothed long short-term memory (FTS-LSTM). This model trains an LSTM autoencoder network to generate future frames on normal video streams, and uses the FTS detector and generation error (GE) detector to detect anomalies on testing video streams. FTS loss is a new indicator in the anomaly–detection area. In the training stage, the model applies a feature trajectory smoothness (FTS) loss to constrain the LSTM layer. This loss enables the LSTM layer to learn the temporal regularity of video streams more precisely. In the detection stage, the model utilizes the FTS loss and the GE loss as two detectors to detect anomalies. By cascading the FTS detector and the GE detector to detect anomalies, the model achieves a high speed and competitive anomaly-detection performance on multiple datasets.

## 1. Introduction

Surveillance cameras are widely used in people’s daily lives. Detecting anomalies in surveillance videos is important for safe-protection and crime prevention. Anomalies in videos generally refer to events that have low probabilities of occurrence [1], or patterns that do not conform to expected behaviors [2].

Abnormal event detection is of great significance in many scenarios. For example, in office areas, illegal intrusion, theft, and fire are anomalies; in transportation scenes, traffic violations and traffic accidents are anomalies [3,4,5]; in public areas, terrorist attacks, robbery, and fare evasion are anomalies. Thus, improving the detection ability of surveillance video in public areas garners attention in research [6,7]. Detecting anomalies in surveillance videos is a challenging task because (1) surveillance videos are private property and (2) anomalous events have rarity, diversity, and scene-dependent properties. It is almost infeasible to gather all kinds of abnormal events and tackle the problem of anomaly detection with a simple classification method [8].

Video anomaly-detection methods can be classified into three categories, i.e., supervised methods, unsupervised methods and semisupervised methods. Supervised methods transform the anomaly-detection task into a binary or multiclassification task, by collecting and annotating a large number of normal and abnormal video samples. Ullah et al. proposed a lightweight model for anomaly detection [9], which works for a real-world surveillance network and employs the residual attention-based long short-term memory (LSTM) which can effectively learn temporal context information and precisely recognize anomalous events. Dubey et al. proposed an innovative framework called DMRMs, which was tested on the UCF–crime and ShanghaiTech datasets [10]. The results and ablation study demonstrated their effectiveness when compared with other methods. The disadvantages of this kind of method include the facts that the workload of sample collection and annotation is huge, and the generalization of detecting unknown abnormal events is poor. The unsupervised method analyzes the distribution of sample space and judges a small number of samples far away from the majority of samples as anomalies. Ionescu et al. proposed a novel framework for abnormal event detection in the video that requires no training sequences [11]. The disadvantages of this kind of method include a large amount of computation, poor real-time performance, and poor anomaly-detection. The semisupervised method transforms anomaly detection into a classification task by only collecting a large number of normal samples. They study the patterns of normal samples and identify those that do not follow normal patterns as abnormal. This kind of method has a small sample collection and sample labeling workload, has good generalization for unknown anomalies, and good real-time anomaly-detection speed. This has gained the most attention among the three kinds of methods.

The semisupervised surveillance video-anomaly detection algorithm has been developed for a long time. Recently, with the excellent performances of deep learning in many computer vision tasks, deep-learning-based semisupervised surveillance video anomaly detection (DSAD) algorithms have gained much attention. These methods use neural networks to learn the manifold distribution of normal samples, and then judge the samples that deviate from the normal manifold distribution as anomalies. Based on the types of indicators in anomaly detection, the semi-supervised methods can be classified into four categories: the deep distance-based method [12,13,14], the deep probability-based method [15,16], the deep generation error-based (GE-based) method [17,18,19,20], and and the aggregation method [21,22,23]. The deep distance-based method clusters samples to multiple groups by the deep neural network (DNN), and judges the samples that are outliers of all normal clusters as anomalies. The deep probability-based method learns the probability distribution of normal video samples, and take samples with low distribution probabilities (DPs) as anomalies. The deep GE-based method trains generative models to generate normal video frames and judge testing frames with large GE errors as anomalies. The aggregation methods train no less than two detectors that belong to the above three methods to detect the video anomaly events.

In the DSAD method, the GE indicator is a very important indicator because of its good anomaly detection and location performances. It usually plays a major role in aggregation methods. In order to improve the anomaly-detection effect of GE, many improvement strategies have been proposed. One important and fundamental improvement strategy is to capture videos’ temporal regularity. In the surveillance video anomaly-detection field, many previous works such as [24,25,26] have proven that LSTM has a solid ability to capture video temporal regularity. These LSTM methods [24,25,26] utilized autoencoder models to generate normal video frames, adopted GE loss to constrain models’ generation performances, and asserted LSTM layers between the encoder and decoder modules to capture videos’ temporal regularity. However, the GE loss does not constrain videos’ features directly, and is not powerful enough to force the maintainance of videos’ temporal regularity in the feature space. Thus, these LSTM methods would not capture videos’ temporal regularity precisely. As a result, the LSTM layer could not effectively improve the anomaly-detection performance of the model. In addition, deep neural networks usually face the problem of large amounts of computation. The way to further reduce the amount of computation and improve the abnormal detection speed of neural networks is a problem that requires constant attention.

In order to solve the aforementioned problems, this paper proposes a new detection model, namely, the feature trajectory-smoothed long short-term memory (FTS-LSTM). In the training stage, the model imposes a temporal smoothing loss on the feature space of the LSTM layer, which enables features to maintain the videos’ temporal regularity better and thus enables the LSTM layer to learn videos’ temporal regularity more precisely. In the detecting stage, the model utilizes the feature-trajectory smoothness (FTS) loss as a new anomaly-detection indicator. The FTS indicator judges frames with high FTS losses as anomalies. It can detect anomalies quickly because of its low computation cost. The generation error (GE) indicator can detect anomalies precisely [19,27]. By cascading the FTS and the GE indicators, the proposed model achieves fast and accurate anomaly-detection performances.

The contributions of the paper are summarized as follows.

- A a video anomaly-detection model, namely, FTS-LSTM, is proposed. In this model, an FTS loss is designed to enable the LSTM layer to learn videos’ temporal regularity better.
- A new indicator to detect anomalies, namely, the FTS indicator, is proposed. It can detect anomalies precisely with a high speed.
- This work has good generalization capability and can easily transfer to other models with LSTM layers.

The overall structure of the article is summarized below. In Section 2, we discuss the development of existing techniques concerning anomaly detection in surveillance videos. Section 3 describes the detail of the novel FTS-LSTM method. In Section 4, the model implementation and experimental results, along with the evaluation of the proposed model are discussed. Finally, the conclusion and future work are given in Section 5.

## 2. Related Work

The development of semisupervised anomaly-detection algorithms can be classified into two stages, namely, the stage of traditional machine learning methods and the stage of deep learning methods. Furthermore, the traditional machine learning methods can be classified into three broad research areas, and the deep learning methods can be classified into four broad research areas.

### 2.1. Traditional Machine Learning Stage

In the traditional machine learning stage, many studies extract features manually and use traditional machine learning models to detect anomalies. Anomaly-detection indicators in this stage can be roughly classified into distance-based (DB) methods, probability-based (PB) methods, and reconstruction error (RE) methods.

The distance-based method [28,29] detects anomalies by using distances from test samples to normal samples or clusters of normal samples. This type of methods usually includes a step of clustering. Before model training, the normal samples are divided into multiple clusters, and then the samples far away from all normal clusters are judged as abnormal. Ionescu et al. [28] used k-means to cluster samples and one-class support vector machines (OC-SVM) to detect outliers. Hinami et al. [29] trained a multitask fast recurrent convolutionary neural network (RCNN) model to extract features. They grouped features into different clusters by k-means and used kernel density estimation (KDE) to detect anomalies on all clusters.

The probability-based method [30,31] learns the distribution probability density of the sample feature space or the inferred relationship between normal features through the model, and then takes the samples with low distribution probability density or those which do not obey the normal inferred relationship as abnormal. Hu X. et al. [32] modeled the distribution of normal sample feature spaces with models in question. They first proposed a local binary pattern feature with a squirrel cage structure, and then modeled the feature space of normal samples with a model in question. Weixin Li et al. [33] used the mixture dynamic texture (MDT) model to construct transition rules for normal sample feature sequences. MDT consists of k-linear dynamic systems, which are used to capture k-state transition laws of normal sample features. When the test sample does not meet any of the normal transition rules, the algorithm judges it as an abnormal event.

The reconstruction error method [34] used the common factors shared by the normal samples to reconstruct normal samples, but abnormal samples cannot be reconstructed because they do not share any common factors. Cong et al. [35] proposed a sparse coding method that weighs word anomalies so that different words have different anomaly weights. Chu et al. [36] proposed a recurrent framework that combines deep feature extraction with sparse coding. They put the module for training 3D convolutional neural networks to extract deep features and the module for learning sparse coding dictionaries with deep features under the same loop framework to be iteratively optimized, so that the features extracted by the network are the features most suitable for the sparse coding method, in order to achieve better performance in terms of good anomaly detection.

### 2.2. Deep Learning Stage

In the deep learning stage, many studies train DNNs to detect anomalies in the end-to-end manner. The indicators can be classified into four categories based on their characters, i.e., the deep distance-based (DDB) method, the deep probability-based (DPB) method, the deep generation error-based (DGE) method, and the aggregation method.

The deep distance-based method [12,13,14] in the deep learning stage clusters samples to multiple groups by DNN in an end-to-end manner. It judges the samples that are outliers of all normal clusters as anomalies. Fan et al. [37] trained a Gaussian mixture fully convolutional variational autoencoder (GMFC-VAE) to map samples to multiple clusters in the latent space and judged samples that have low condition probabilities with any existing clusters as anomalies. Wu et al. [14] trained a deep one-class neural network (DeepOC) to map normal samples into a single hypersphere and judged the samples mapped out of the hypersphere as anomalies.

The deep probability-based method [20,38,39] learns the probability distribution of normal videos and judges samples with low distribution probabilities as anomalies. It uses the discriminator to output the DPs of the video frames to detect anomalies. Ravanbakhsh et al. [39] trained two GANs to generate motion images from appearance images which were generated from motion images. They combined two DP score maps generated by two discriminators to detect anomalies.

The deep generation error-based method [17,18,19,20,22,24,25,26,40,41,42,43] trains generative models to generate normal video frames and judges testing frames with large GE errors as anomalies. Hasan et al. [26] first introduced the autoencoder(AE) to video anomaly detection. Gong et al. [40] proposed a memory-augmented autoencoder (MemAE) to limit the AE’s generalization ability. Zhou et al. [41] proposed an attention-driven training loss to alleviate the imbalance problem between the foreground and stationary background. In order to capture videos’ spatiotemporal regularity, many methods [18,21,22,24,25,42,43] have utilized the LSTM-AE to detect anomalies. There are some works which train no less than two detectors to disclose the video anomaly events which belongs to deep generation error-based method.

The aggregation method [21,22,23] trains no less than two detectors to disclose the video anomaly events. Lee et al. proposed a spatiotemporal adversarial network to detect anomalies [21]. The algorithm extracts two anomaly detectors which are a generative error detector and a generative adversarial network (GAN) probabilistic detector. The two detectors disclose anomalies with a weighted sum of the anomaly scores of the two detectors. Wang et al. proposed an integrated approach called primary–auxiliary fusion [23]. The core detector is a video anomaly detector based on the pixel generation error, and the auxiliary detector is a detector with high accuracy in detecting strong normality and strong anomaly. The algorithm extracts this decision ability from the auxiliary detector and weighs it with the outlier score in the main detector to obtain an integrated detector.

## 3. Method

**Figure 1:**
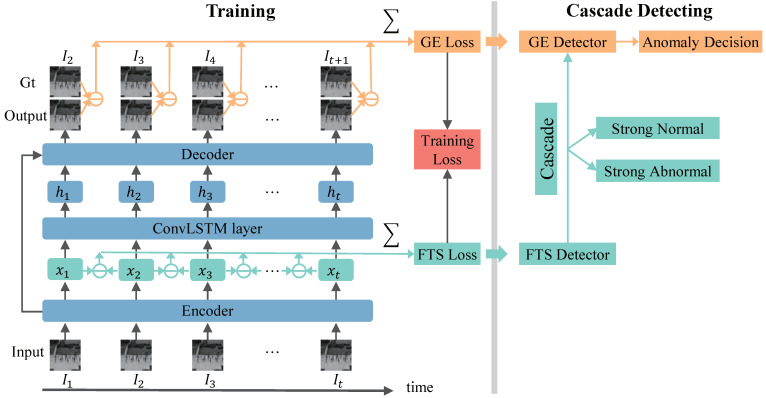
Pipeline of the proposed method. FTS-LSTM trains an LSTM-AE to predict future frames for input frames. FTS-LSTM uses two losses to constrain the model: a GE loss and a FTS loss. The GE loss enables the model to predict future frames precisely. The FTS loss enables features to maintain videos’ temporal regularity. In the testing period, the FTS loss and the GE loss as indicators are utilized to detect anomalies. FTS-LSTM cascades the FTS indicator and the GE indicator to achieve fast and accurate performances.

The pipeline of the proposed work is illustrated in Figure 1. It uses normal videos to train the model and detect anomalies in the testing videos. This section introduces the proposed work in three aspects, i.e, the network structure, the training process, and the detecting process.

### 3.1. Network Structure

As shown in Figure 1, the proposed method consists of three network modules, which are the encoder module, the ConvLSTM module, and the decoder module, repectively. There is a skip connection from the encoder to the decoder, which can improve the model ability to transmit more information from the encoder to the decoder.

#### 3.1.1. Encoder Module

The encoder module extracts spatial features for input frames. It consists of several 2D spatial convolution layers. Let E express the encoder, and $\left\{I_{1},\ldots ,I_{t},\ldots ,I_{T}\right\}$ be *T* consecutive input video frames. The feature of the frame $I_{t}$ can be represented as

(1) $$x_{t}=E\left(I_{t}\right),$$

where $x_{t}$ is the extracted feature for frame $I_{t}$. Therefore, we can get *T* consecutive features $\left\{x_{1},\ldots ,x_{t},\ldots ,x_{T}\right\}$ for $\left\{I_{1},\ldots ,I_{t},\ldots ,I_{T}\right\}$.

#### 3.1.2. ConvLSTM Module

**Figure 2:**
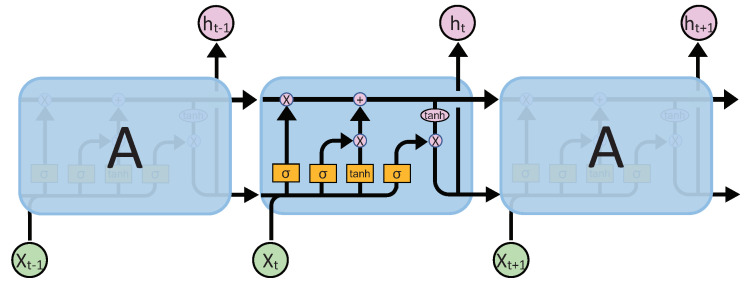
LSTM structure.

The ConvLSTM module aims to capture videos’ temporal regularities in the feature space. The ConvLSTM is widely used in many video processing tasks. The process of the ConvLSTM module can be expressed as

(2) $$\begin{array}{cc} {\hat{C}}_{t} & =relu(W_{C}⊙\left[h_{t-1},x_{t}\right]+b_{C}) \end{array}$$

(3) $$\begin{array}{cc} i_{t} & =\sigma (W_{i}⊙\left[h_{t-1},x_{t}\right]+b_{i}) \end{array}$$

(4) $$\begin{array}{cc} f_{t} & =\sigma (W_{f}⊙\left[h_{t-1},x_{t}\right]+b_{f}) \end{array}$$

(5) $$\begin{array}{cc} C_{t} & =f_{t}*C_{t-1}+i_{t}*{\hat{C}}_{t} \end{array}$$

(6) $$\begin{array}{cc} o_{t} & =\sigma (W_{o}⊙\left[h_{t-1},x_{t}\right]+b_{o}) \end{array}$$

(7) $$\begin{array}{cc} h_{t} & =o_{t}*relu\left(C_{t}\right), \end{array}$$

where $i_{t},f_{t}$ and $o_{t}$ are the input gate, forget gate, and output gate at time *t*; ${\hat{C}}_{t}$ is the input information of the LSTM at time *t*; $C_{t}$ is the cell state at time *t* (it stores the information of history frames $\left[I_{T-4},I_{T-1}\right]$); $h_{t}$ is the output of the LSTM layer at time *t*; $W_{C},W_{i},W_{f},W_{o}$ are the weights metrics; $b_{C},b_{i},b_{f},b_{o}$ are the biases of ConvLSTM; ⊙ and ∗ represent the convolution operation and pointwise multiplication, respectively; and σ and relu represent the sigmoid and ReLU [44] activation function. The LSTM network is shown in Figure 2. We use H to represent the ConvLSTM module. At time *t*, the ConvLSTM’s processing function can be simply expressed as

(8) $$h_{t}=H\left(x_{t},h_{t-1}\right),$$

where $x_{t}$ is the input at time *t*; $h_{t-1}$ is the hidden state at time t−1; and $h_{t}$ is the hidden state at time *t*. Based on (8), we get *T* consecutive hidden states $\left\{h_{1},\ldots ,h_{t},\ldots ,h_{T}\right\}$ for consecutive features $\left\{x_{1},\ldots ,x_{t},\ldots ,x_{T}\right\}$.

#### 3.1.3. Decoder Module

The decoder module plays the role of a generator. It predicts future frames for input frames given $\left\{h_{1},\ldots ,h_{t},\ldots ,h_{T}\right\}$. It consists of several 2D convolution layers and 2D deconvolution layers. We utilize D to express the decoder, and use ${\hat{I}}_{t+1}$ to represent the prediction result for frame $I_{t}$. We have

(9) $${\hat{I}}_{t+1}=D\left(h_{t}\right),$$

where D is the decoder and ${\hat{I}}_{t+1}$ is the output of D, whose ground truth is $I_{t+1}$.

### 3.2. The Training Process

In the training process, we use a GE loss and an FTS loss to constrain the model to learn videos’ normal regularity.

#### 3.2.1. The GE Loss

The GE loss consists of two sub-GE losses, $l_{int}$ and $l_{gdl}$, whose functions are represented as follows,

(10) $$\begin{array}{cc} L_{GE} & =l_{int}+l_{gdl}, \end{array}$$

(11) $$\begin{array}{cc} l_{int} & =\sum_{t=1}^{T}{∥{\hat{I}}_{t+1}-I_{t+1}∥}_{2}, \end{array}$$

(12) $$\begin{array}{cc} l_{gdl} & =\sum_{t=1}^{T}({∥\nabla_{x}\left({\hat{I}}_{t+1}\right)-\nabla_{x}\left(I_{t+1}\right)∥}_{1}+∥\nabla_{y}\left({\hat{I}}_{t+1}\right)-\nabla_{y}\left(I_{t+1}\right){∥}_{1}), \end{array}$$

where $l_{int}$ is the intensity loss, which is applied to penalize the losses on pixels’ intensities; $l_{gdl}$ is the gradient loss which is applied to penalize errors around edges; and $\nabla_{x}$ and $\nabla_{y}$ represent the spatial derivatives along the *x*-axis and *y*-axis, respectively.

The purpose of GE loss is to enable the model to accurately generate normal samples. It does not constrain videos’ features directly, because there is a decoder module between the feature space and the GE loss. As a result, the GE loss is not powerful enough to force features maintaining videos’ temporal regularity, and the LSTM layers would not capture videos’ temporal regularity precisely.

#### 3.2.2. FTS Loss

In order to capture the videos’ temporal regularity precisely, we present an FTS loss to constrain the feature space directly. The content of the video frames changes smoothly over time. Therefore, the features of video frames should also change smoothly in the feature space.

Based on this point, we design the FTS loss to force temporal-consecutive features to be similar. We use the Euclidean distance to measure the similarity between features and accumulate the distances between all temporal-neighbored features to formulate the FTS loss. The FTS loss is expressed as

(13) $$L_{FTS}=\sum_{t=1}^{T-1}{∥x_{t+1}-x_{t}∥}_{2}.$$

#### 3.2.3. Global Training Loss

We combine the GE loss and FTS loss to train the model. The global training loss has a coefficient that is called λ, and it can be represented as

(14) $$\begin{array}{c} L_{train}=L_{GE}+\lambda *L_{FTS}. \end{array}$$

### 3.3. Detecting Process

In the detecting period, we design a GE detector and FTS detector based on the GE loss and the FTS loss, respectively. We cascade these two detectors to achieve faster and better anomaly detections.

This section first introduces the GE detector’s and the FTS detector’s working mechanisms, then analyses why the FTS loss is helpful to improve GE detector’s anomaly-detection performance.

#### 3.3.1. The GE Detector

The model is trained to predict normal samples. It cannot predict anomalous samples well. We use the $l_{int}$ of the last frame to detect anomalies. Considering that anomalies usually occur in local areas, the maximum of block-level GEs in a frame is used to detect anomalies [45], which is defined as

(15) $$\begin{array}{c} {GE}_{map}\left(t\right)=\sum_{c}{∥{\hat{I}}_{t+1}-I_{t+1}∥}_{2}, \end{array}$$

(16) $$\begin{array}{c} S_{GE}\left(t\right)=max\left(mean_{bl\_size}\left({GE}_{map}\left(t\right)\right)\right), \end{array}$$

where ${GE}_{map}\left(t\right)$ is the GE map of the predicted frame ${\hat{I}}_{t+1}$; $S_{GE}\left(t\right)$ is the anomaly score for frame $I_{t+1}$ in the GE detector; $mean_{bl\_size}$ indicates a mean filter with kernel size bl_size; and *c* indicates the number of channels of a frame.

#### 3.3.2. The FTS Detector

The DNN learns the mapping function between two manifold distributions, which is only applicable to samples that obey the manifold distributions. When a sample does not obey the input manifold distribution, its mapping position will deviate from its target position on the output distribution. We call the difference between the mapping position of the sample and the target mapping position as a mapping error. In FTS-LSTM, the encoder learns a mapping function from the manifold of normal frames to a feature space. When an abnormal sample (outliers of the normal manifold) is input to the encoder, there will be a large number of mapping errors in the feature space, and anomalous videos FTS loss will increase. Therefore, the FTS loss can be used to detect abnormalities. Based on this point, we use the FTS loss as an indicator to detect anomalies and judge the samples with large FTS losses as anomalies. Considering that anomalies occur in local areas, we use the maximum value of the FTS loss map to detect anomalies. The FTS detector is defined as

(17) $$\begin{array}{cc} {FTS}_{map}\left(t\right)= & \sum_{c}{∥x_{t}-x_{t-1}∥}_{2}, \end{array}$$

(18) $$\begin{array}{cc} S_{FTS}\left(t\right)= & max({FTS}_{map}\left(t\right)), \end{array}$$

where ${FTS}_{map}\left(t\right)$ is the FTS-loss-map of $I_{t}$; $S_{FTS}\left(t\right)$ is the anomaly score for $I_{t}$ in the FTS detector; and *c* indicates the number of channels of the feature map.

As shown in (17), the FTS detector detects anomalies by detecting the difference between the apparent characteristics of the target over time. Therefore, the detector is suitable to detecting dynamic anomalies (the abnormal targets having motion in the scene).

#### 3.3.3. Cascade

The FTS detector detects anomalies in the feature space. It is faster than the GE detector. The FTS detector can be cascaded with the GE detector to detect anomalies. When a sample is input into the model, its features are extracted and then the SN and SA samples are detected with the FTS detector. Afterward, the remaining features are fed to the following network modules and the GE detector is used to make the final decision. In the cascading process, it is essential to set suitable thresholds for FTS detector In this paper, we set the SA threshold ${thr}^{a}$ and the SN threshold ${thr}^{n}$ based on the FTS anomaly scores of the training data. We have

(19) $$\begin{array}{cc} {thr}^{a} & =max\left(S_{FTS}^{train}\right)+\left(max\left(S_{FTS}^{train}\right)-min\left(S_{FTS}^{train}\right)\right)*\gamma_{a}, \end{array}$$

(20) $$\begin{array}{cc} {thr}^{n} & =min\left(S_{FTS}^{train}\right)+\left(max\left(S_{FTS}^{train}\right)-min\left(S_{FTS}^{train}\right)\right)*\left(1-\gamma_{n}\right), \end{array}$$

where max(scores) and min(scores) indicates the maximum value and the minimum value of the scores, respectively; $S_{FTS}^{train}$ indicates the FTS anomaly scores of the training data; $\gamma_{a}$ and $\gamma_{n}$ indicate the strict coefficients for ${thr}^{a}$ and ${thr}^{n}$, respectively.The higher the $\gamma_{a}$ and $\gamma_{n}$, the more credible the extracted SA and SN samples. Generally, $\gamma_{a}$ and $\gamma_{n}$ are in the range of [0,1].

As shown in (19), we set the maximum value of normal training samples’ FTS loss, $max(S_{FTS}^{train})$, as the base value of the SA threshold. We added the second term, ($max\left(S_{FTS}^{train}\right)-min\left(S_{FTS}^{train}\right))*\gamma_{a}$, as the strengthen value. The strengthen value is calculated by the max–min difference value multiplying a ratio. As shown in (20), we set the minimum value of normal training samples’ FTS loss, $min(S_{FTS}^{train})$, as the base value of the SN threshold. It is too strict to detect SN samples. Therefore, we added the second term, $\left(max\left(S_{FTS}^{train}\right)-min\left(S_{FTS}^{train}\right)\right)*\left(1-\gamma_{n}\right)$, as the relaxing value. The relaxing value is calculated by the $\left(max\left(S_{FTS}^{train}\right)-min\left(S_{FTS}^{train}\right)\right)$ difference value multiplying a ratio.

#### 3.3.4. Discussion

The GE detector can detect both temporal and spatial anomalies in videos. Its anomaly-detection mechanism is analyzed as follows. Let us substitute Equations (8) and (9) into Equation (16). Then the GE detector can be expressed as

(21) $$\begin{array}{cc} S_{GE} & =max(mean\left(\sum_{c}{|D\left(H\left(h_{t-1},x_{t}\right)\right)-I_{t+1}|}^{2}\right)). \end{array}$$

As shown in (21), the GE is generated by ${\hat{I}}_{t+1}$ and ${\hat{I}}_{t+1}$ is generated from $h_{t}$. The $h_{t}$ has two information sources: the $x_{t}$ and the $h_{t-1}$.

The $x_{t}$ supplies the spatial information of the current input frame $I_{t}$. It is generated by the encoder module. The encoder module is trained to extract spatial features for normal frames; it cannot extract features correctly for abnormal frames. Therefore, there will be information differences between the extracted features and the aiming features for abnormal frames. The information differences in $x_{t}$ will lead to the large GEs in ${\hat{I}}_{t+1}$. Therefore, the GE loss can be used to detect spatial anomalies.

The $h_{t-1}$ supplies history information including $I_{t-4},I_{t-3},I_{t-2},I_{t-1}$, respectively. The $h_{t-1}$ captures history information by the memory cell $C_{t}$ and three gates $i_{t},f_{t},o_{t}$ in the LSTM module. In the training process, the memory cell and three gates are trained to capture information from sequences of historical features that obey normal temporal regularities. When features do not obey normal temporal regularities, the three gates will capture incorrect information from historical features. Thus, there will be errors of information in $h_{t-1}$. The error of information in $h_{t-1}$ will lead to the larger GE losses in ${\hat{I}}_{t+1}$. Therefore, the GE loss can be used to capture temporal anomalies.

As analyzed above, the better the LSTM layer learns normal videos’ temporal regularity, the better the performance the GE detector can capture videos’ temporal anomalies. The better FTS loss enables feature space to maintain normal videos temporal regularity, the better the LSTM layer can learn videos’ temporal regularity. Therefore, the FTS loss can help the GE detector to achieve better anomaly-detection performances.

## 4. Results

In this section, we carry out experiments to demonstrate the effectiveness of the proposed method.

### 4.1. Datasets

We evaluate our method on three popular public datasets.

UCSD dataset [46] has two subdatasets: The UCSD Pedestrian 1 (Ped1) dataset and the UCSD Pedestrian 2 (Ped2) dataset. The Ped1 dataset contains 34 training videos and 36 testing videos. The Ped2 dataset contains 16 training videos and 12 testing videos. The two datasets are captured from different scenarios. Their abnormal events include cycling, skateboarding, crossing lawns, cars, etc. These two subdatasets are usually used separately.

The CUHK Avenue dataset [34] contains 16 training videos and 21 testing videos. The abnormal events include running, throwing schoolbag, throwing papers, etc. The size of people may change with the positions and angles of the camera.

The ShanghaiTech (SH) dataset [19] contains 330 training videos and 107 testing videos. The videos are captured from 13 different scenes. The abnormal events include running, cars, throwing schoolbag, etc.

### 4.2. Implementation Details

In all experiments, video frames are resized to 256×256 pixels, the pixel values of video frames are normalized to [−1,1], the LSTM layer’s length T=5,minibatch=2, and λ=100. In the training process, the Adam algorithm [47] is utilized as the optimizer. Each dataset trains for 200,000 iterations with minibatch=2 on a single GTX 1080 GPU. The learning rate is set $1\times 10^{-4}$ when the iteration is low than 40,000, which is set to $1\times 10^{-5}$ when the iteration is high than 40,000. In the testing stage, set $bl\_size=30,\gamma_{a}=0.2$. In Ped1 and Ped2 datasets, $\gamma_{n}=0.8$. In Avenue and SH datasets, $\gamma_{n}=0.4$ to achieve better performances.

**Figure 3:**
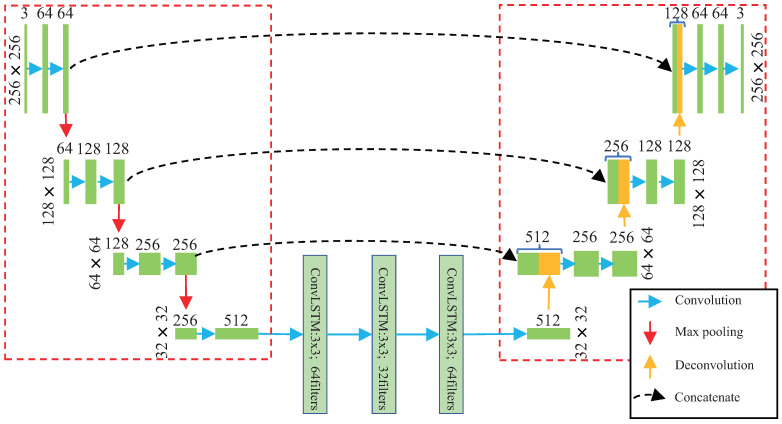
The detail of the network structure of our work. There are three zones in the network, in which the left zone is called the encoder, the right zone is called the decoder, and the rest of the structure in the middle is the LSTM network.

The detail of FTS-LSTM network is shown in Figure 3. All the kernel sizes and strides of the convolution layers are (3,3) and (1,1), respectively. All the kernel sizes and strides of the transpose convolution layers are (2,2) and (2,2), respectively. The pool size and strides of the polling layers are (2,2) and (2,2), respectively. We adopt the Relu activation function in all convolution layers. The green rectangles indicate the tensor obtained by the convolution operation, and the orange rectangles indicate the tensor obtained by deconvolution. In the deconvolution process, the number of tensor channels is halved, and the height and width of tensors are doubled. The function of concatenate is to transmit more information from the encoder to the decoder so that the decoder can obtain a better generation effect and better anomaly-detection effect [8].

As shown in Figure 3, The entire network contains 21 layers of convolution or deconvolution operations: seven layers of 3×3 convolution operations in the encoder module, three layers of 3×3 convolution operations in the LSTM module, three deconvolution operations in the Decoder network, and eight convolution operations in the decoder network.

### 4.3. Evaluation Metric

In video anomaly detection, the most commonly used evaluation metric is the receiver operation characteristic (ROC) curve and the area under this curve (AUC). A higher AUC value indicates better anomaly-detection performance. This paper adopts the frame-level AUC to evaluate anomaly-detection performances.

### 4.4. Anomaly-Detection Performances

Table 1 shows anomaly detection ROC/AUC performances of the proposed model, comparing with some state-of-the-art (SOTA) and classic methods, including DDB [14], DPB [20], DGE [8,19,40,41,48], and the aggregation methods [21,22,23]. In the Table, the optimal performance in each dataset is marked with bold font, and the suboptimal performance is marked with bold italic font. The proposed model achieves optimal and suboptimal performances on Ped2, Avenue, and SH datasets. Meanwhile, its detection speed is 117 FPS on average, which is far faster than other algorithms. These performances demonstrate the superiority of the proposed method.

**Figure 4:**
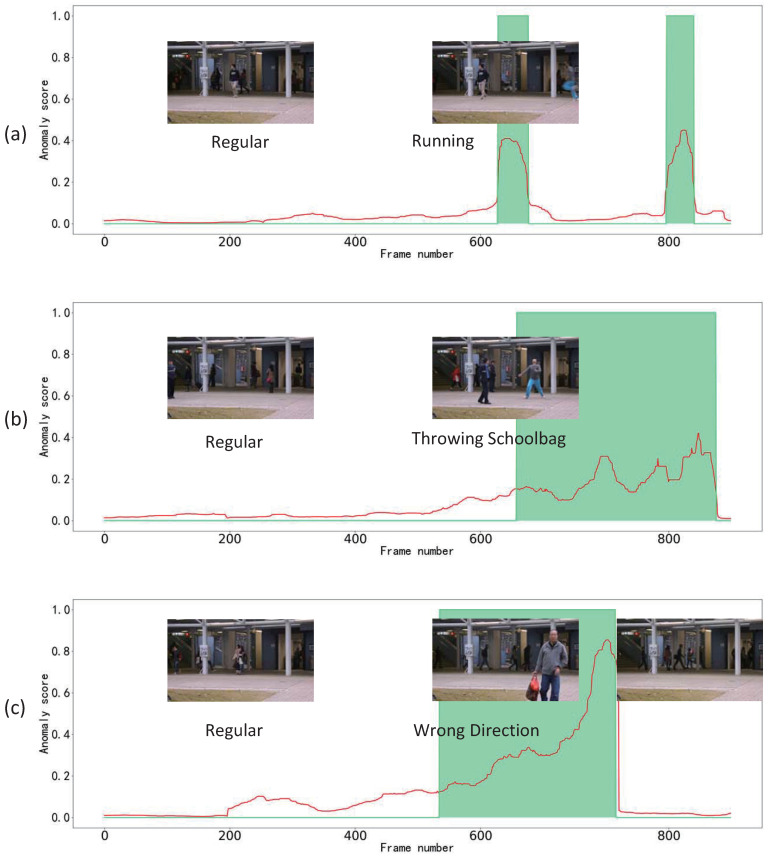
Frame-level anomaly-detection scores (between 0 and 1) provided by our FST-LSTM framework based on the late fusion strategy, for test in the Avenue dataset. The green lines and green zone represent the ground truth abnormal events. The red lines represent our scores. (**a**) Test video 4 in the Avenue dataset. (**b**) Test video 5 in the Avenue dataset. (**c**) Test video 6 in the Avenue dataset.

**Figure 5:**
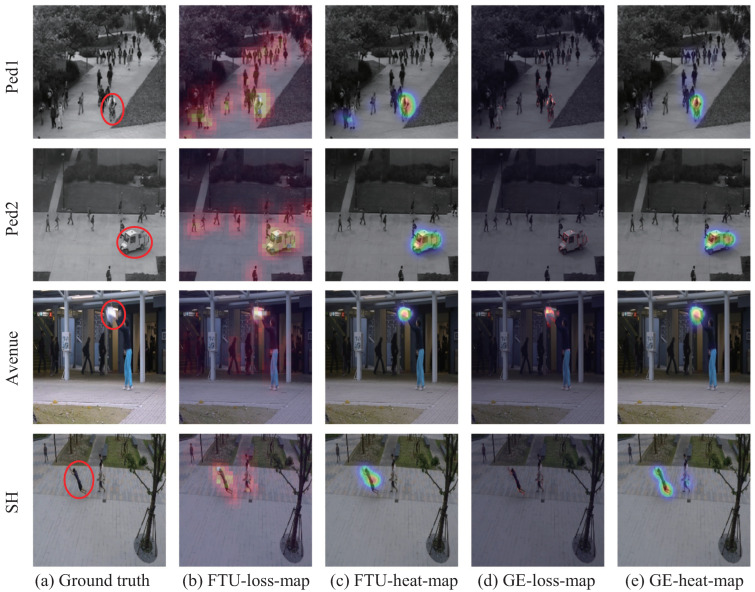
Anomaly-detection visualization. (**a**) Anomalous frames in different datasets. The contents in red circles are anomalous events. (**b**) FTS loss’s intensity map. (**c**) FTS loss’s heatmap. (**d**) GE loss’s intensity map. (**e**) GE loss’s heatmap.

Frame-level anomaly-detection scores (between 0 and 1) provided by our FST-LSTM framework are shown in Figure 4. The cyan zone represents the ground-truth abnormal events and our scores are illustrated in red. The pictures in the figure are the frames of the Avenue dataset captured from test video 4 to test video 6, which illustrate the effect of our framework. Anomaly-detection heatmaps of videos are shown in Figure 5. As shown in Figure 5b,c, the FTS loss in anomalous areas are higher than that in normal areas. They demonstrate that the FTS loss can detect and localize anomalies. Figure 5d,e show intensity maps and heatmaps of the GE indicator. They demonstrate the anomaly-detection performances of the GE indicator.

### 4.5. Ablation Study

This section carries out experiments to demonstrate the problems proposed in the introduction and prove the effectiveness of the proposed model in solving these problems.

#### 4.5.1. Feature Space TSNE Visualization

**Figure 6:**
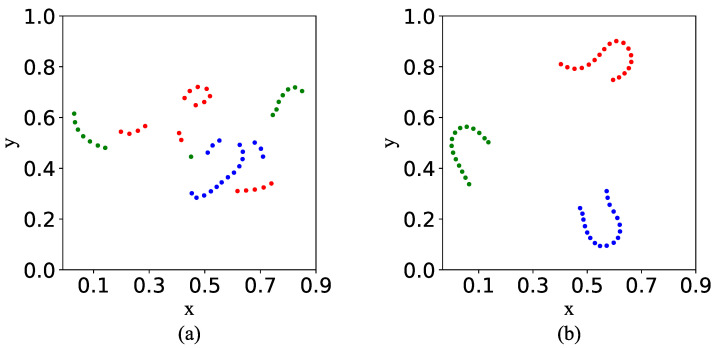
Dots with different colors indicates features belonging to different videos. (**a**) Without FTS loss. (**b**) With FTS loss.

Figure 6 visualizes two video features in the model’s feature space. As shown in Figure 6a, when the model is trained without utilizing the FTS loss, video features are randomly distributed in the feature space. It indicates that the feature space does not maintain videos’ temporal regularity precisely. As shown in Figure 6b, when the model is trained with utilizing the FTS loss, video features are distributed in the feature space in an orderly manner. The features of different videos are separable from each other. It indicates that the model’s feature space maintained videos’ temporal regularity. The visualization verified the effectiveness of the FTS loss on maintaining videos’ temporal regularity. The result demonstrates the proposed model can solve the question when utilizing LSTM layer to detect anomalies.

**Table 1:** Frame-level ROC/AUCs of different methods. The bold font represent the optimal performance, and the bold italic font represent the suboptimal performance.

Method	–	Ped1	Ped2	Avenue	SH	Speed
Deep Distance-based	DeepOC [14]	83.5	96.9	86.6	–	***40 FPS***
Deep Probability-based	Tang et al. [20]	84.7	96.3	85.1	71.5	30 FPS
Aggregation methods	STAN [21]	82.1	96.5	87.2	–	–
	TAM-Net [22]	83.5	98.1	78.3	–	–
	MAAS [23]	**85.8**	**99.0**	**92.1**	69.7	4 FPS
Deep Generation-error-based	Unet [8]	83.1	95.4	85.1	72.8	12 FPS
	Ts-Unet [48]	–	97.8	88.4	–	12 FPS
	sRNN [19]	–	92.2	83.5	69.6	10 FPS
	MemAE [40]	–	94.1	83.3	71.2	38 FPS
	Zhou et al. [41]	83.9	96.0	86.0	–	–
	FTS-LSTM (ours)	83.5	***98.3***	***91.1***	***72.9***	**117 FPS**

#### 4.5.2. Impact of FTS Loss on the GE Detector

**Figure 7:**
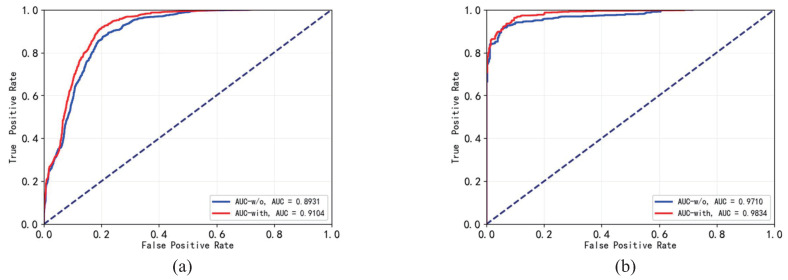
The ROC/AUC curves of the GE detectors trained with and without utilizing the FTS loss on multiple datasets. The red curve represents the detector trained with FTS loss. The blue curve represents the detector trained without FTS loss. (**a**) The ROC/AUC curves on Avenue dataset. (**b**) The ROC/AUC curves on Ped2 dataset. The dashed blue line represent the ROC curve of a completely random classifier.

The FTS loss enables LSTM layer to learn videos’ temporal regularity more precisely. It increases GE detector’s anomaly-detection performance. Table 2 and Figure 7 show the anomalous frames’ GE saliencies in models trained with and without utilizing the FTS loss and shows the ROC/AUCs of corresponding models. The table demonstrates that the FTS loss improves anomalous frames’ GE saliencies and improves GE detector’s anomaly-detection performances.

#### 4.5.3. Impact of the FTS Loss on FTS Detector

The DNN trained on normal samples cannot maintain relationships among abnormal samples. Table 3 calculates the FTS loss saliencies of anomalous frames compared with normal frames. As shown in the table, all the FTS loss anomaly saliencies are positive, which indicates that the FTS losses of the anomalous frames are higher than that of the normal frames. It indicates that the FTS loss can be used to detect anomalies, which proves our analysis.

**Figure 8:**
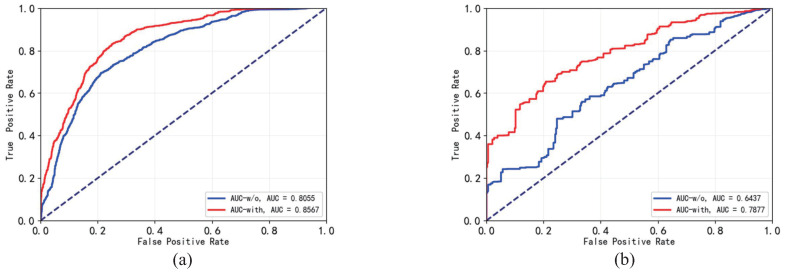
The ROC/AUC curves of the FTS detectors trained with and without utilizing the FTS loss on multiple datasets. The red curve is represents the detector trained with FTS loss. The blue curve represents the detector trained without FTS loss. (**a**) The ROC/AUC curves on Avenue dataset. (**b**) The ROC/AUC curves on Ped2 dataset. The dashed blue line represent the ROC curve of a completely random classifier.

Table 3 and Figure 8 show anomaly-detection performances of the FTS detectors. The FTS loss strengthened the encoder to maintain more relationships among normal frames. It increased the anomaly saliencies of the anomalous frames in FTS.

#### 4.5.4. Detection Speed Analysis

By cascading the FTS and GE detectors, the proposed model achieves fast and precise performances. Table 4 shows anomaly-detection ROC/AUCs and speeds of different detectors. It demonstrates that, by cascading the FTS and the GE detectors, the model maintains GE detector’s ROC/AUC and achieves a faster speed than the GE detector.

As shown in Table 4, this work can achieve a speed of 117 FPS, and this high detection speed mainly benefits from the low computational complexity of the FTS detector. The FTS detector only calls the encoder module of the network (7 layers 3×3 convolution operations) to detect anomalies and can filter out most video frames in anomaly detection. Only a small number of video frames are transmitted to the subsequent network module, which greatly reduces the amount of calculation in the anomaly-detection process.

#### 4.5.5. Impact of Weight λ

**Figure 9:**
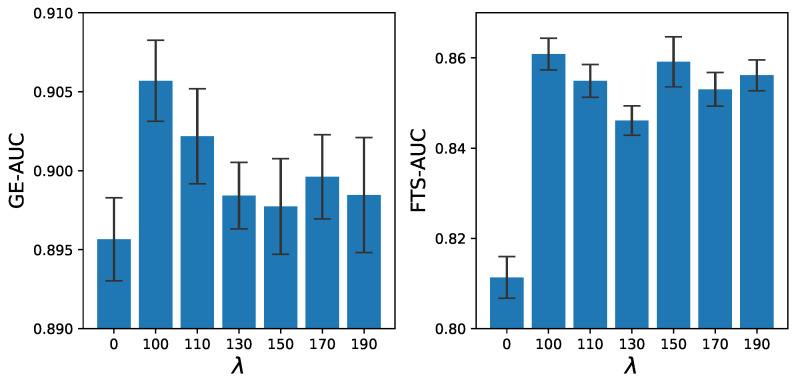
Frame-level ROC/AUCs of the GE and FTS detectors under different FTS loss weights.

Figure 9 shows the anomaly-detection ROC/AUC of GE metrics and FTS metrics under different λ. This figure proves that the FTS loss can robustly improve the anomaly-detection performance of the model.

#### 4.5.6. Generality

Table 5 shows anomaly-detection saliency and ROC/AUC with or without applying FTS loss in the LSTM model [24]. The anomaly-detection performance and anomaly saliency of the the LSTM model have been significantly improved with FTS loss. This result proves that the temporal smoothing loss in the feature space is general for improving the anomaly-detection performance of the generative model by restraining generated errors.

### 4.6. Limitation

As described above, our proposed method achieves relatively better performance on the UCSD dataset and ShanghaiTech dataset. However, this method might not be good at detecting static anomaly time. For example, the car parked on the sidewalk, the FTS can detect the object in to scene but cannot respond to the static car out because the target brings no changes to the frame’s apparent feature. Generally, abnormal events occur along with a dynamic process. Therefore, this limitation is acceptable to surveillance video anomaly detection.

## 5. Conclusions

This paper proposes a FTS-LSTM method for video anomaly detection. It trains a LSTM-AE to generate normal videos and to detect anomalies. In the training process, it uses the FTS loss and the GE loss to constrain the model. In the detecting process, it cascades the FTS and the GE indicators to detect anomalies. Experiments on multiple datasets reveal the proposed method’s effectiveness and efficiency. The shortcoming of the FTS indicator is that it cannot detect static anomalies. In general monitoring scenarios, the occurrence of abnormal events generally have a dynamic process. Therefore, this shortcoming can be ignored. In the future, we will combine the FTS loss with Transformer and the GRU method to explore the proposed method’s generalization, and we will study the solution of combining the FTS detector with a static anomaly-detection method to improve the algorithm’s ability.

## Figures and Tables

**Table 2:** Frame-level GE saliency and ROC/AUCs of the GE detectors on multiple datasets. The bold font represent GE saliency of anomalous frames and ROC/AUC performances utilizing the FTS loss.

	FTS Loss	Ped1	Ped2	Avenue	SH
GE saliency of	w/o	1.930	3.657	2.645	1.184
Anomalous frames	with	**2.205**	**3.985**	**2.656**	**1.366**
ROC/AUC	w/o	82.73	97.10	89.31	71.20
	with	**83.51**	**98.34**	**91.04**	**72.92**

**Table 3:** Frame-level FTS saliency and ROC/AUCs of the FTS detectors on multiple datasets. The bold font represent FTS saliency of anomalous frames and ROC/AUC performances utilizing the FTS loss.

	FTS Loss	Ped1	Ped2	Avenue	SH
FTS saliency of	w/o	0.086	0.055	0.342	0.342
Anomalous frames	with	**0.162**	**0.122**	**0.639**	**0.374**
ROC/AUC	w/o	64.02	64.37	80.55	67.22
	with	**70.22**	**78.77**	**85.67**	**68.71**

**Table 4:** Frame-level ROC/AUCs of the cascaded detector on multiple datasets.

	ROC/AUC				Speed
	Ped1	Ped2	Avenue	SH	
FTS Detector	70.22	78.77	85.67	68.71	186 FPS
GE Detector	83.51	98.34	91.04	72.92	50 FPS
Cascade	83.51	98.34	91.14	72.92	117 FPS

**Table 5:** Saliency and ROC/AUC of the LSTM model with or without applying FTS loss. The bold font represent saliency of anomalous frames and ROC/AUC performances utilizing the FTS loss.

	FTS Loss	Ped2	Avenue	Average
Saliency of Anomalous frames	w/o	0.9278	1.086	1.0007
	with	**1.104**	**1.192**	**1.148**
ROC/AUC	w/o	76.51	79.18	77.85
	with	**82.25**	**81.62**	**81.94**

## Data Availability

Not applicable.
